# Pharmacokinetics, safety, and population pharmacokinetic profiles of Ritanine^®^ in Chinese subjects with impaired or normal liver function: a Phase I trial

**DOI:** 10.3389/fphar.2026.1833170

**Published:** 2026-06-01

**Authors:** Qingmei Li, Jiajia Mai, Min Wu, Hong Zhang, Xinyu Yang, Yuanyuan Huang, Cuiyun Li, Jianyuan Yin

**Affiliations:** 1 School of Pharmaceutical Sciences, Jilin University, Jilin, China; 2 Department of Pediatric Nephrology, Children’s Medical Center, The First Hospital of Jilin University, Jilin, China; 3 Phase I Clinical Research Center, The First Hospital of Jilin University, Jilin, China; 4 Jiangsu Hengrui Pharmaceuticals Co., Ltd., Lianyungang, Jiangsu, China; 5 Fujian Shengdi Pharmaceutical Co., Ltd., Xiamen, Fujian, China

**Keywords:** clinical trial, hepatic impairment, HR20013, pharmacokinetics, rolapitant, safety

## Abstract

**Objectives:**

Ritanine (HR20013) is a fixed-dose combination of fosrolapitant (218 mg, a rolapitant prodrug) and palonosetron (0.25 mg), designed to enhance rolapitant’s solubility and reduce allergic reactions associated with intravenous administration. Metabolized primarily by the liver, hepatic impairment may affect its pharmacokinetics (PK) and safety.

**Methods:**

This nonrandomized, open-label, single-dose study evaluated the effect of moderate hepatic impairment on Ritanine’s PK and safety. Eight subjects with moderate hepatic impairment and eight healthy controls each received a single intravenous infusion.

**Results:**

Compared with healthy controls, the geometric least-squares mean ratios for C_max_, AUC_0-t_, and ${\text{AUC}}_{0-\infty }$ in subjects with moderate hepatic impairment were 1.27, 1.51, and 1.51 for fosrolapitant; 0.61, 1.15, and 1.24 for rolapitant; 0.74, 0.85, and 1.13 for metabolite M19; and 0.90, 1.77, and 1.46 for palonosetron, respectively. Hepatic impairment did not significantly alter the T_max_ or elimination half-life (t_1/2_) of the above components. Ritanine exhibited good safety and tolerability in this study.

**Conclusion:**

Moderate hepatic impairment has a minimal impact on the pharmacokinetics of Ritanine, suggesting that no dosage adjustment is necessary for patients with moderate hepatic impairment in clinical practice.

**Clinical Trail Registration:**

http://www.chinadrugtrials.org.cn, identifier CTR20233771.

## Introduction

Chemotherapy-induced nausea and/or vomiting (CINV) affects the quality of life of patients and reduces their compliance with anti-tumor treatment, thereby affecting the therapeutic effect (Zhang et al., 2018; Hsu et al., 2021; Patel et al., 2022; Kennedy et al., 2024; Albanell-Fernández et al., 2025; Jin et al., 2025). Currently, the commonly used antiemetic drugs in clinical practice can be roughly classified into 5-HT3 receptor antagonists, NK-1 receptor antagonists, and glucocorticoids, etc (Navari, 2006; Razvi et al., 2019; Wang et al., 2021; Herrstedt et al., 2024). The NK-1 receptor antagonist rolapitant was approved by the FDA for marketing in 2015 under the trade name VARUBI. Rolapitant has poor water solubility, and an injectable emulsion was subsequently developed. However, due to drug-related allergic reactions and anaphylactic shock, this dosage form was voluntarily withdrawn from the market in 2018, possibly because of sensitization caused by the emulsion excipients.

To address the aforementioned shortcomings of rolapitant, a mixed formulation of fosrolapitant—the prodrug of rolapitant—and palonosetron (PALO) was developed and named Ritanine (code-named HR20013) (Zhao et al., 2025). This reduces the number of drug administrations for patients and enhances their medication compliance (Wang et al., 2017; Aapro et al., 2021; Aapro et al., 2022). Ritanine has demonstrated good safety and tolerability in both healthy subjects and cancer patients (Chen et al., 2021; Zhao et al., 2025). The Phase III clinical trial results demonstrate that Ritanine in combination with dexamethasone is noninferior to the regimen of fosaprepitant plus palonosetron and dexamethasone in preventing cisplatin-based highly emetogenic chemotherapy-induced nausea and vomiting. Additionally, it exhibits favorable tolerability and holds promise for reducing the impact of such symptoms on patients’ daily activities (Zhou et al., 2025). On 29 May 2025, Ritanine was initially approved in China for the prevention of both acute and delayed nausea and vomiting associated with highly emetogenic chemotherapy in adult patients.

*In vitro* studies indicate that palonosetron is metabolized primarily by CYP2D6, with minor contributions from CYP3A and CYP1A2. However, CYP2D6 phenotype (poor vs. extensive metabolizer) does not meaningfully alter its clinical pharmacokinetics. Hepatic impairment also has no clinically significant effect on palonosetron clearance; therefore, no dose adjustment is needed in patients with any degree of liver dysfunction (Helsinn Healthcare, 2003). Fosrolapitant is rapidly and completely converted into the active metabolite rolapitant *in vivo* via amide hydrolysis and is primarily metabolized by CYP3A4 to produce the metabolite M19. Hepatic impairment may alter these processes, thereby affecting the drug’s safety and efficacy. Therefore, this study evaluated the impact of moderate hepatic impairment on the plasma exposure of Ritanine’s components and their metabolites, as well as on the safety and tolerability of Ritanine in patients with moderate hepatic impairment.

## Subjects and methods

### Study design

This study adopted a single-center, non-randomized, open-label, parallel-group, single-dose trial design to evaluate the safety and pharmacokinetic characteristics of Ritanine for injection in subjects with moderate hepatic impairment. Two groups were enrolled: the moderate hepatic impairment group (Child–Pugh class B) and the healthy control group, each consisting of 8 subjects. All subjects received an intravenous infusion of Ritanine (containing fosrolapitant 218 mg and palonosetron 0.25 mg) over a period of 1 h (±10 min). Clinical Trial Registration Number: CTR20233771.

Healthy subjects were matched to individuals with hepatic impairment according to sex, as well as body weight (within ±10 kg of the group average) and age (within ±10 years of the group average). Screening evaluations were conducted within 1 week before drug administration. Qualified participants completed baseline assessments and were admitted to the study facility on Day −1. All subjects received intravenous infusion of the study drug at Day 1. Discharge occurred on Day 3, with follow-up safety evaluations performed on Days 3, 11, and 36. The study protocol and informed consent documentation were reviewed and approved by the Ethics Committee of the First Hospital of Jilin University. The trial was conducted in accordance with the ethical principles of the Declaration of Helsinki, Good Clinical Practice guidelines for drug trials, and all relevant regulatory standards and legal requirements in China. Written informed consent was obtained from all participants before any study-related procedures.

### Subjects

Chinese male or female subjects who met the following inclusion criteria were eligible for participation in the study: aged 18–65 years; body mass index (BMI) of 18–32 kg/m^2^; creatinine clearance rate of at least 60 mL/min; and willingness to adhere to strict contraceptive measures from the screening phase through 6 months after the final dose of the investigational drug. In addition, subjects with hepatic impairment were required to fulfill the following supplementary criteria: chronic liver disease caused by primary hepatic disorders (e.g., hepatitis B, hepatitis C, autoimmune hepatitis, or alcoholic liver disease) with liver function classified as Child–Pugh class B.

Exclusion criteria: Subjects with a history of hypersensitivity to rolapitant or palonosetron; those who have used CYP2D6 or CYP3A4 enzyme inducers or inhibitors within 1 month before screening; and those with a screening QTcF interval > 470 msec in males or > 480 msec in females. Additional exclusion criteria for subjects with hepatic impairment: alanine aminotransferase (ALT) or aspartate aminotransferase (AST) > 10 × upper limit of normal (ULN); absolute neutrophil count (ANC) < 0.75 × 10^9^/L; hemoglobin < 60 g/L; and alpha-fetoprotein (AFP) > 100 ng/mL. Additional exclusion criteria for the healthy control group: abnormal findings on physical examination, laboratory tests, or abdominal ultrasound that, in the investigator’s judgment, are clinically significant.

### Pharmacokinetics (PK) analysis

PK blood sample collection times: within 2 h before the start of intravenous infusion; at 0.5, 1, 2, 4, 6, 12, 24, 48, 144, 240, 336, 504, 672, 840, and 1,008 h after the start of infusion; and immediately after the end of infusion. Blood sample collection times for free fraction assessment: at 1, 2, 240, and 504 h after the start of administration. A volume of 4 mL of venous blood was collected at each time point into EDTA-K2 anticoagulant-containing vacutainer tubes. After collection, samples were centrifuged at 1,500 g for 10 min within a temperature range of 2 °C–8 °C to isolate plasma. Quantitative analysis of fosrolapitant, rolapitant, its metabolite M19, and palonosetron in plasma was performed using validated liquid chromatography-tandem mass spectrometry (LC-MS/MS) methods at Suzhou Haike Pharmaceutical Technology Co., Ltd., following predefined acceptance criteria for bioanalytical sample assessment. The calibration curves for these analytes covered concentration ranges of 8–4,000 ng/mL for fosrolapitant, 8–4,000 ng/mL for rolapitant, 1.00–300 ng/mL for M19, and 0.0200–10.0 ng/mL for palonosetron. The corresponding lower limits of quantification (LLOQ) were established as 8 ng/mL, 8 ng/mL, 0.05 ng/mL, and 0.02 ng/mL, respectively.

### Safety assessments

Safety evaluations included monitoring of adverse events, physical examinations, vital signs, laboratory parameters, and electrocardiograms (ECGs). The intensity of adverse events was graded according to the National Cancer Institute’s Common Terminology Criteria for Adverse Events (CTCAE) version 5.0. Treatment-emergent adverse events (TEAEs) and treatment-related adverse events (TRAEs) were actively monitored throughout the 2-day inpatient period at the study facility and during subsequent follow-up visits up to Day 36 post-dose.

### Development of the population PK model

Population PK analysis of fosrolapitant and rolapitant was performed simultaneously with Phoenix NLME software (Version 8.1, Certara). A one- or two-compartment model with first-order elimination model was tested as the structural model. Interindividual variability was assumed to be log normally distributed and modeled using multiplicative exponential random effects. Residual random effects were described with a multiplicative model. Goodness-of-fit diagnostics, visual predictive check with 500 replicates and bootstrap with 500 replicates were used to evaluate the model and obtain CIs for parameter estimates. The success rate of bootstrap was 100%.

### Statistical analysis

Pharmacokinetic data for fosrolapitant, rolapitant, its metabolite M19, and palonosetron were processed using Phoenix WinNonlin software (version 8.3). Non-compartmental analysis was applied to determine key pharmacokinetic parameters based on actual blood sampling times. Which included peak plasma concentration (C_max_), observed time at Cmax (T_max_), terminal elimination half-life (t_1/2_), area under the plasma concentration-time curve from time zero to infinity ( ${\text{AUC}}_{0–\infty }$ ), area under the curve from time zero to the last quantifiable concentration (AUC_0–t_), clearance (CL), and volume of distribution (V).

The natural logarithm-transformed values of C_max_, ${\text{AUC}}_{0–\infty }$ , and AUC_0–t_ for each analyte were subjected to an analysis of variance (ANOVA) model, with treatment group included as a fixed effect, to estimate the geometric mean ratio and corresponding 90% confidence interval (CI) comparing the moderate hepatic impairment group to the healthy control group. Demographic, safety, and pharmacokinetic data were summarized using descriptive statistics. Continuous variables are presented as mean ± standard deviation, while categorical variables are reported as frequency counts and percentages. All statistical analyses were conducted using SAS software (version 9.4).

## Results

### Subject demographics

A total of 16 subjects were enrolled, and all completed the study, with a mean age of 52.5 years, a mean weight of 67.35 ± 7.36 kg, and a mean body mass index of 24.63 ± 2.64 kg/m^2^. Demographic characteristics were well balanced between the hepatic impairment and healthy control groups (Table 1).

**TABLE 1:** Demographic and baseline characteristics of this study.

Baseline parameter	Moderate hepatic impairment (N = 8)	Healthy control (N = 8)
Age, years	54.9 (5.33)	51.8 (4.30)
Sex (N (%), male)	7 (87.5)	7 (87.5)
Ethnicity (N (%), Han)	7 (87.5)	8 (100.0)
Weight, kg	68.11 (9.572)	66.59 (4.797)
BMI, kg/m^2^	25.16 (3.242)	24.10 (1.943)

Data are mean (standard deviation), unless stated otherwise.

### Pharmacokinetics

#### Fosrolapitant

**FIGURE 1:**
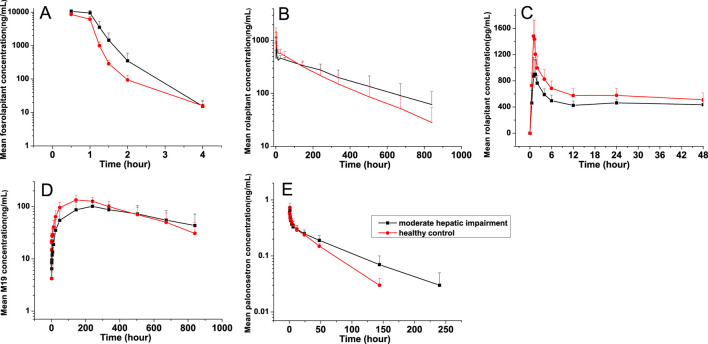
Plasma concentration–time profiles of fosrolapitant, rolapitant, M19, and palonosetron in subjects with moderate hepatic impairment and in the healthy control group. **(A)** fosrolapitant; **(B)** rolapitant; **(C)** The PK curve of rolapitant within 0–48 h after administration; **(D)** M19; **(E)** palonosetron; Data are presented as the mean (± standard deviation).

After administration of fosrolapitant, subjects in both groups exhibited rapid elimination. The median T_max_ was consistent between the two groups (0.5 h). The geometric mean ratios (90% CI) of C_max_, AUC_0-t_ and ${\text{AUC}}_{0-\infty }$ for fosrolapitant in the moderate hepatic impairment group relative to the healthy control group were 1.27 (1.12–1.43), 1.51 (1.29–1.76), and 1.51 (1.29–1.76), respectively; the corresponding t_1/2_ were 0.42 h and 0.64 h. Fosrolapitant exposure was approximately 50% higher in subjects with moderate hepatic impairment than in the healthy control group; however, both groups rapidly converted fosrolapitant to the active metabolite rolapitant. The unbound fraction of fosrolapitant in plasma was similar between the two groups (approximately 0.5%) (Tables 2, 3; Figure 1).

**TABLE 2:** Pharmacokinetic parameters of fosrolapitant, rolapitant, and palonosetron following single-dose administration (N = 8 per group; mean (%CV)).

Parameter	Fosrolapitant		Rolapitant		Palonosetron	
	Moderate hepatic impairment	Healthy control	Moderate hepatic impairment	Healthy control	Moderate hepatic impairment	Healthy control
C_max_ (ng/mL)	10740.00 (12.9)	8487.50 (15.0)	949.38 (26.9)	1522.50 (18.6)	0.67 (14.2)	0.74 (18.2)
T_max_ ^*^ (hour)	0.50 (0.50, 1.00)	0.50 (0.50, 0.50)	1.25 (1.00, 2.00)	1.00 (1.00, 1.25)	1.00 (0.50, 1.00)	1.00 (0.50, 1.00)
AUC_0-t_ (h × ng/mL)	10298.30 (20.1)	6758.89 (13.7)	170208.06 (29.0)	147851.50 (30.5)	31.18 (34.1)	17.25 (26.4)
${\text{AUC}}_{0-\infty }$ (h × ng/mL)	10307.42 (20.1)	6773.86 (13.7)	201065.62 (37.3)	158820.39 (33.7)	34.89 (32.6)	22.72 (5.8)
t_1/2_ (hour)	0.42 (17.0)	0.64 (10.5)	264.80 (48.4)	189.40 (41.0)	81.80 (36.9)	44.32 (16.7)
V (L)	13.64 (40.4)	30.44 (19.1)	​	​	839.62 (10.5)	709.33 (21.7)
CL (L/h)	21.98 (22.1)	32.72 (13.7)	​	​	7.92 (35.0)	11.04 (5.9)

* Median (Minimum value, Maximum value).

**TABLE 3:** Geometric mean ratios (90% confidence interval) of Cmax and AUC for moderate hepatic impairment versus healthy control, derived from analysis of variance (ANOVA).

​	Geometric mean ratios (90% confidence interval)			
PK parameters	Fosrolapitant	Rolapitant	M19	Palonosetron
C_max_	1.27 (1.12–1.43)	0.61 (0.50–0.75)	0.74 (0.59–0.94)	0.90 (0.78–1.04)
AUC_0-t_	1.51 (1.29–1.76)	1.15 (0.86–1.55)	0.85 (0.69–1.06)	1.77 (1.34–2.35)
${\text{AUC}}_{0-\infty }$	1.51 (1.29–1.76)	1.24 (0.87–1.77)	1.13 (0.76–1.66)	1.46 (1.11–1.94)

The median T_max_ of rolapitant was 1.00–1.25 h in both groups. The C_max_ of rolapitant in the moderate hepatic impairment group was approximately 39% lower than that in the healthy control group, with the geometric mean ratio (90% CI) being 0.61 (0.50–0.75). However, exposure to rolapitant was comparable to that in the healthy control group, with geometric mean ratios (90% CI) of AUC_0-t_ and ${\text{AUC}}_{0-\infty }$ being 1.15 (0.86–1.55) and 1.24 (0.87–1.77), respectively. The unbound fraction of rolapitant in plasma was similar between the two groups (approximately 0.1%) (Tables 2, 3; Figure 1).

The C_max_, AUC_0-t_ and ${\text{AUC}}_{0-\infty }$ of the rolapitant metabolite M19 in the moderate hepatic impairment group were comparable to those in the healthy control group, with geometric mean ratios (90% CI) of 0.74 (0.59–0.94), 0.85 (0.69–1.06), and 1.13 (0.76–1.66), respectively. The median T_max_ of M19 was delayed in the moderate hepatic impairment group (240 h) compared with the healthy control group (192 h). The unbound fraction of M19 in plasma was similar between the two groups (approximately 0.2%) (Supplementary Table S1; Table 3; Figure 1).

#### Palonosetron

The median Tmax of palonosetron was consistent between the two groups, occurring at 1 h after administration. In subjects with moderate hepatic impairment, no difference in C_max_ was observed compared with the healthy control group; however, AUC_0-t_ and ${\text{AUC}}_{0-\infty }$ were increased, with geometric mean ratios (90% CI) relative to the healthy control group of 1.77 (1.34–2.35) and 1.46 (1.11–1.94), respectively. CL was decreased and t_1/2_ was prolonged in subjects with moderate hepatic impairment. CL values were 7.92 L/h and 11.04 L/h, and t_1/2_ values were 81.80 h and 44.32 h in the moderate hepatic impairment and healthy control groups, respectively (Tables 2, 3; Figure 1).

#### Safety and tolerability

A total of 5 subjects (62.5%) in the healthy control group experienced TEAEs, among whom 4 subjects (50.0%) experienced TRAEs. A total of 6 subjects (75.0%) in the moderate hepatic impairment group experienced TEAEs, among whom 2 subjects (25.0%) experienced TRAEs. Except for one subject (12.5%) in the moderate hepatic impairment group who experienced grade 3 neutrophil count reduction, lymphocyte count reduction, and white blood cell count reduction, the severity of all other TRAEs was grade 1 or 2. One subject experienced a single grade 4 serious adverse event (SAE; platelet count reduction) that was unrelated to the study drug. No adverse events resulted in death, discontinuation of study drug administration, or study withdrawal. The overall safety and tolerability of a single intravenous infusion of Ritanine injection were favorable in this study (Table 4).

**TABLE 4:** Treatment-emergent adverse events (TEAEs) by hepatic function group (n (%))

Adverse events	Moderate hepatic impairment (N = 8)	Healthy control (N = 8)
The number of subjects with at least one adverse event	6 (75.0)	5 (62.5)
Injection site reaction	0 (0.0)	1 (12.5)
Injection site pain	0 (0.0)	2 (25.0)
Positive urine red blood cells	2 (25.0)	0 (0.0)
Detection of urine protein	2 (25.0)	0 (0.0)
Decreased neutrophil count	1 (12.5)	0 (0.0)
Decreased lymphocyte count	1 (12.5)	0 (0.0)
Decreased white blood cell count	1 (12.5)	0 (0.0)
Decreased platelet count	1 (12.5)	0 (0.0)
Increased serum creatinine	1 (12.5)	1 (12.5)
Increased serum glucose	0 (0.0)	1 (12.5)
Ventricular premature contractions	1 (12.5)	0 (0.0)
Rash	2 (25.0)	0 (0.0)
Petechiae	1 (12.5)	0 (0.0)

## Discussion

Ritanine is a fixed-dose combination therapy comprising fosrolapitant—an emerging prodrug that acts as a neurokinin-1 (NK-1) receptor antagonist—and palonosetron, a second-generation 5-hydroxytryptamine-3 (5-HT3) receptor antagonist. This combination is under investigation for the prevention of chemotherapy-induced nausea and vomiting.

In this study, after administration of 218 mg fosrolapitant, exposure to fosrolapitant—assessed as AUC—in subjects with moderate hepatic impairment was approximately 50% higher than that in the healthy control group. However, exposure to its active metabolite rolapitant—assessed as AUC—was comparable to that in the healthy control group, whereas the Cmax was approximately 39% lower. These findings are consistent with those from the hepatic impairment study of the approved rolapitant tablet (VARUBI®) (Wang et al., 2018), which showed that the C_max_ of rolapitant in subjects with moderate hepatic impairment was approximately 25% lower than that in healthy subjects, whereas no significant difference in AUC was observed. Based on the results of this study, it is hypothesized that hepatic impairment may slightly reduce the rate of conversion of fosrolapitant to rolapitant but does not significantly affect the extent of conversion.

A two-compartment disposition model with first-order elimination and a multiplicative error model was used to simultaneously fit the plasma concentrations of fosrolapitant and rolapitant in the population PK study (Supplementary Table S2; Supplementary Figures S1–S4). Due to the small sample size, the metabolite M19 was excluded from the model, and no formal covariate analysis was performed. This study conducted exploratory structural model evaluation and residual diagnostics, and assessed the impact of hepatic impairment on the clearance of fosrolapitant and rolapitant. Hepatic impairment grade significantly affected fosrolapitant clearance: clearance in subjects with hepatic impairment was 2.9 L/h lower than that in healthy subjects, whereas rolapitant clearance was comparable between the two groups (Supplementary Figure S5) (Wang et al., 2018). These findings are consistent with the changes in fosrolapitant clearance observed in the NCA of this study. Furthermore, the unbound fractions of fosrolapitant, rolapitant, and M19 in plasma were all <1% and similar between the two groups, suggesting that moderate hepatic impairment does not affect plasma protein binding of these compounds (Wang et al., 2018).

After administration of 0.25 mg palonosetron, the geometric mean AUC_0-t_ values of palonosetron were 29.59 and 16.69 hng/mL in subjects with moderate hepatic impairment and in the healthy control group, respectively, with a geometric mean ratio of approximately 1.77. Compared with the PK data from healthy subjects in the previous Phase 1 study conducted in China, the AUC_0-t_ was approximately 30 hng/mL (Grunberg and Koeller, 2003; Chen et al., 2021). The palonosetron AUC in subjects with moderate hepatic impairment in this study was comparable to that reported in previous studies (difference < 25%), whereas the palonosetron AUC in the healthy control group was lower than that reported in previous studies—potentially attributable to interindividual variability. Based on all available pharmacokinetic data from healthy subjects, no significant difference in palonosetron exposure was observed between subjects with moderate hepatic impairment and the healthy control group. In addition, the original clinical pharmacology study of palonosetron hydrochloride injection (ALOXI®) demonstrated that hepatic impairment did not significantly affect the systemic clearance of this product relative to the healthy control group. Furthermore, results from the Phase III clinical trials of ALOXI®(Aapro et al., 2006; Wu et al., 2018) showed that the primary efficacy endpoint—complete response within 24 h after chemotherapy—was comparable between the 0.25 mg and 0.75 mg palonosetron dose groups, and the increased exposure did not translate into additional safety risks.

The overall safety profile of Ritanine injection was favorable and well tolerated. The incidence of TEAEs and TRAEs was similar between the moderate hepatic impairment group and the healthy control group. The most commonly reported adverse reactions associated with the approved oral formulation of rolapitant include neutropenia, decreased appetite, dizziness, dyspepsia, hiccups, abdominal pain, and others—predominantly mild to moderate in severity (Heo and Deeks, 2017; Rapoport, 2017; Zhao et al., 2025; Zhou et al., 2025). The most commonly reported adverse reactions associated with palonosetron include headache, constipation, diarrhea, dizziness, fatigue, and abdominal pain—predominantly mild to moderate in severity (Chen et al., 2021; Aapro et al., 2022; Kim et al., 2022). In this study, one subject in the moderate hepatic impairment group developed Grade 3 neutropenia (baseline: 2.11 × 10^9^/L; nadir: 0.88 × 10^9^/L on Day 3; recovery: 2.07 × 10^9^/L on Day 5). A causal relationship with hepatic impairment could not be excluded; however, no other subjects experienced the adverse reactions listed above. The fixed-dose combination of fosrolapitant (218 mg) and palonosetron (0.25 mg) did not increase the incidence of adverse reactions associated with either component administered alone—and was associated with a numerically lower incidence of adverse reactions overall.

This study is a single-center study that only included 8 patients with moderate hepatic impairment and corresponding healthy controls, which is an exploratory study. There may be certain biases when extrapolating its conclusion to a broader population with hepatic impairment. Subsequent real-world studies should expand the sample size, include more patients with hepatic impairment, and further analyze the relationship between the dosage and drug concentration to support dose optimization. Clinical real-world data of patients with hepatic impairment should be continuously collected to provide a basis for dose adjustment.

## Conclusion

Ritanine exhibited favorable safety and tolerability profiles in both individuals with moderate hepatic impairment and healthy subjects. The observed pharmacokinetic variations between them are not considered clinically significant, and therefore do not necessitate routine dose modifications in patients with moderate hepatic impairment.

## Data Availability

The raw data supporting the conclusions of this article will be made available by the authors, without undue reservation.
