# Supplementary material for: Pharmacokinetics, safety, and population pharmacokinetic profiles of Ritanine® in Chinese subjects with impaired or normal liver function: a Phase I trial
Source: Front Pharmacol. 2026 Jun 1;17:1833170. doi: 10.3389/fphar.2026.1833170 (PMC13266129; doi:10.3389/fphar.2026.1833170)
Supplement: Supplementary file 1 [file Supplementaryfile1.docx]

**Supplement**


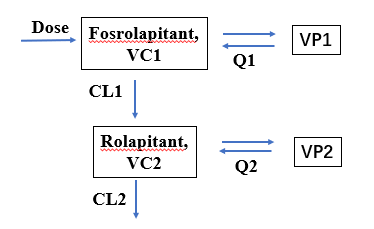


**Supplementary Figure 1. Schematic diagram of the population pharmacokinetic model structure for fosrolapitant and rolapitant.**


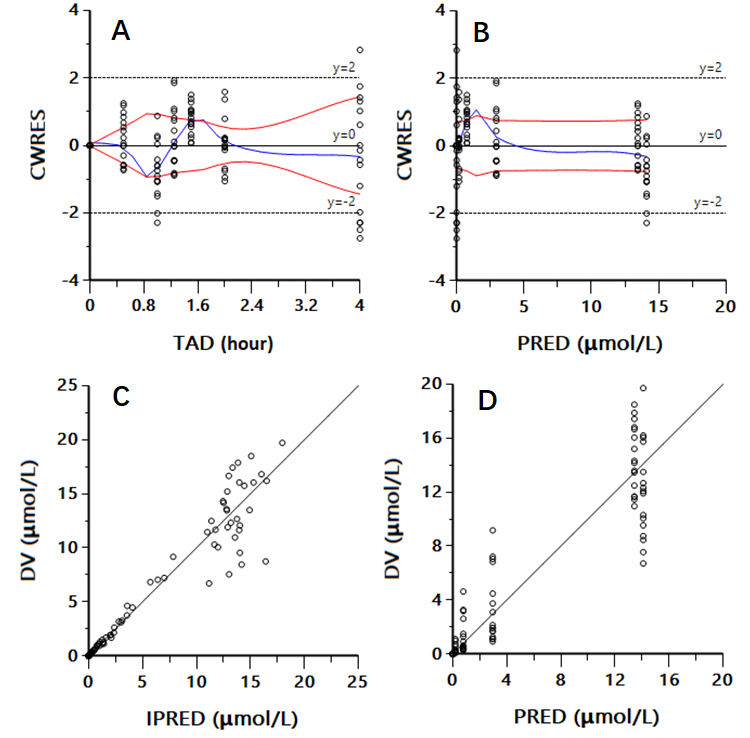


**Supplementary Figure 2. Goodness-of-fit plots of the population pharmacokinetics model of fosrolapitant.** (A) Conditional weighted residual (CWRES) vs. time after dose (TAD); (B) CWRES vs. population prediction (PRED); (C) Observed drug concentration (DV) vs. individual prediction (IPRED); (D) DV vs. PRED; The black line is the line of unity or the zero reference line, and the blue line is the result of locally weighted scatterplot smoothing, and the red line is its 95% CI.


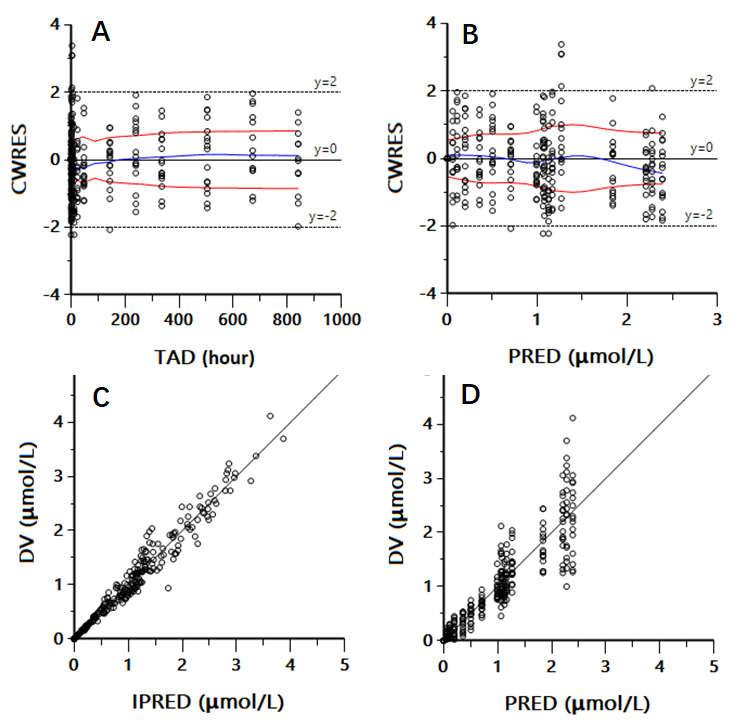


**Supplementary Figure 3. Goodness-of-fit plots of the population pharmacokinetics model of** **rolapitant.** (A) Conditional weighted residual (CWRES) vs. time after dose (TAD); (B) CWRES vs. population prediction (PRED); (C) Observed drug concentration (DV) vs. individual prediction (IPRED); (D) DV vs. PRED; The black line is the line of unity or the zero reference line, and the blue line is the result of locally weighted scatterplot smoothing, and the red line is its 95% CI.


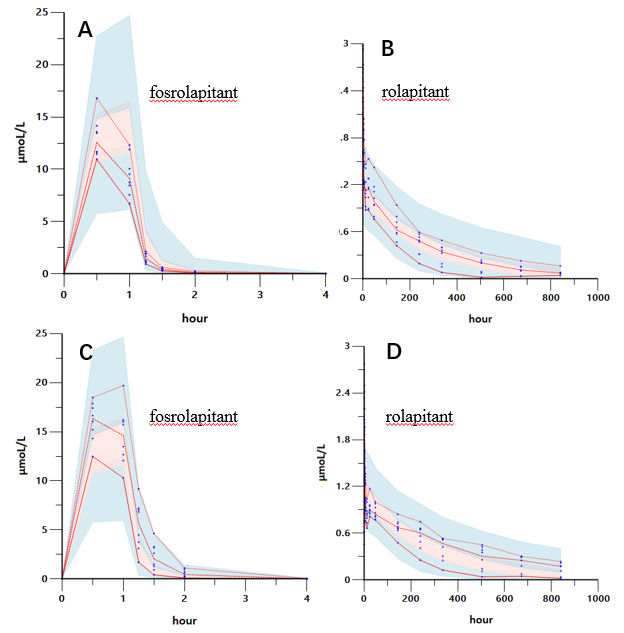


**Supplementary Figure 4. Visual predictive check of the final population pharmacokinetic model.** (A, B) Healthy controls; (C, D) Moderate hepatic impairment.

The blue dots represent the observed concentration values; the three red curves (from bottom to top) correspond respectively to the 5th, 50th, and 95th percentiles of the observed values; the colored shaded areas represent the 90% prediction intervals for the corresponding predicted percentiles.

Due to the small sample size, some of the 5th, 50th, and 95th percentiles of the observed concentrations fall within the 90% prediction intervals of the corresponding predicted percentiles; nonetheless, the overall trends remain broadly consistent. This study aims to preliminarily verify model feasibility and represents the first attempt to jointly develop a population pharmacokinetic model for rolapitant and its prodrug fosrolapitant, thereby laying the foundation for subsequent formal modeling.


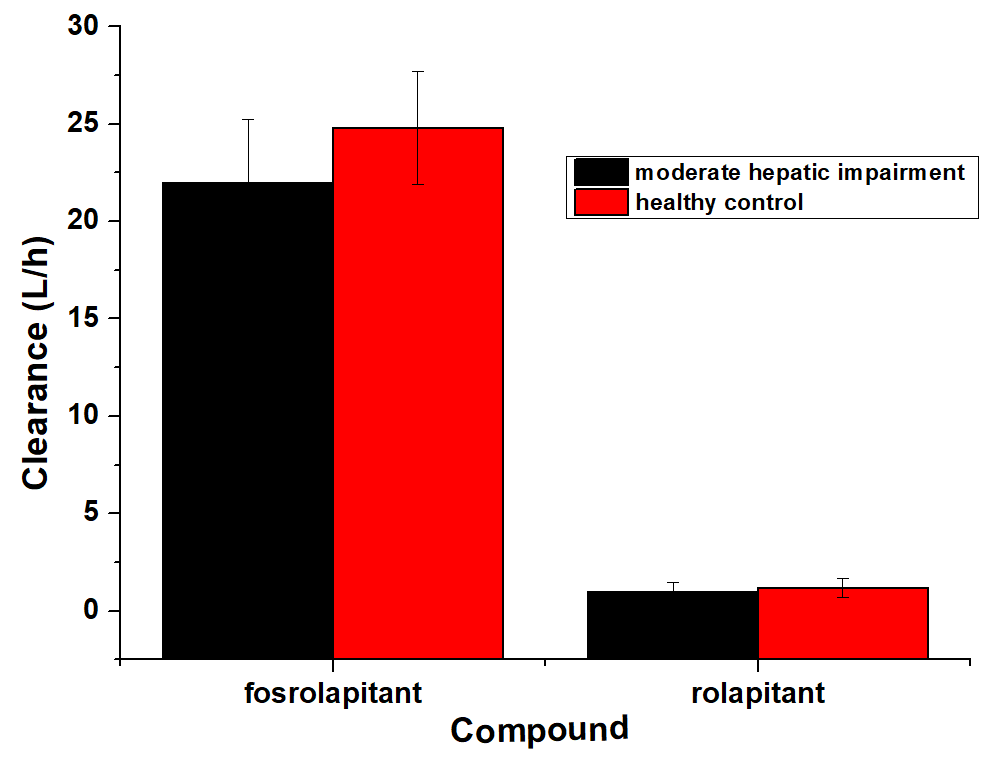


**Supplementary Figure 5.** The clearance of fosrolapitant and rolapitant in the population PK model.

**Supplementary** **Table 1. Pharmacokinetic parameters of M19 following single-dose administration (n=8 for each group, Mean (%CV))**

| Parameter | moderate hepatic impairment | healthy control |
| --- | --- | --- |
| C_max_ (ng/mL) | 106.74 (20.9) | 139.11 (15.9) |
| T_max_^*^ (hour) | 240.00 (143.92, 504.08) | 192.13 (144.18, 335.67) |
| AUC_0-t_ (h×ng/mL) | 58324.04 (24.9) | 67715.36 (20.0) |
| AUC_0-∞_ (h×ng/mL) | 102430.79 (49.3) | 85607.66 (30.6) |
| t_1/2_ (hour) | 708.81 (114.6) | 308.36 (65.3) |

*: Median (Minimum value, Maximum value)

**Supplementary** **Table 2. Parameter estimates of the final population pharmacokinetic model of fosrolapitant and rolapitant**

| Parameter | Estimate | RSE% | Bootstrap | |
| --- | --- | --- | --- | --- |
|  |  |  | Mean | 95% CI |
| Fixed effect |  |  |  |  |
| CL1 (L/h) | 23.146 | 7.111 | 23.326 | 20.428-26.528 |
| Q1 (L/h) | 0.932 | 11.970 | 0.948 | 0.738-1.168 |
| CL2 (L/h) | 0.998 | 9.722 | 1.014 | 0.852-1.242 |
| Q2 (L/h) | 59.085 | 15.959 | 59.139 | 41.052-79.992 |
| VC1 (L) | 3.648 | 4.678 | 3.657 | 3.294-4.057 |
| VP1 (L) | 0.889 | 14.420 | 0.905 | 0.677-1.149 |
| VC2 (L) | 94.995 | 9.349 | 95.032 | 81.318-112.405 |
| VP2 (L) | 185.336 | 6.005 | 186.664 | 163.621-210.216 |
| Random effect |  |  |  |  |
| CL1 | 0.024 | 44.559 | 0.022 | 0-0.045 |
| CL2 | 0.161 | 26.592 | 0.154 | 0.069-0.239 |
| VC1 | 0.115 | 40.021 | 0.109 | 0.026-0.192 |
| VC2 | 0.147 | 29.561 | 0.132 | 0.056-0.208 |
| Residual error |  |  |  |  |
| Stdev1 | 0.213 | 8.280 | 0.208 | 0.171-0.242 |
| Stdev2 | 0.137 | 5.655 | 0.134 | 0.116-0.15 |

The number 1 in the parameter name indicates that this parameter pertains to fosrolapitant, while the number 2 indicates that it relates to rolapitant; CL, clearance of central compartment; VC, the central compartment; Q, clearance between central compartment and peripheral compartment; VP, the peripheral compartment;
